# Social isolation, social support and loneliness as predictors of cardiovascular disease incidence and mortality

**DOI:** 10.1186/s12877-021-02602-2

**Published:** 2021-12-13

**Authors:** Rosanne Freak-Poli, Joanne Ryan, Johannes T. Neumann, Andrew Tonkin, Christopher M. Reid, Robyn L. Woods, Mark Nelson, Nigel Stocks, Michael Berk, John J. McNeil, Carlene Britt, Alice J. Owen

**Affiliations:** 1 Department of Epidemiology and Preventive Medicine, School of Public Health and Preventive Medicine, Monash University, 553 St Kilda Road, 3004 Melbourne, Victoria, VIC Australia; 2 Department of Cardiology, University Heart and Vascular Center Hamburg, Hamburg, Germany; 3 German Center for Cardiovascular Research (DZHK), Partner Site Hamburg/Kiel, Lübeck, Germany; 4 School of Public Health, Curtin University, 6102 Perth, WA Australia; 5 Menzies Institute for Medical Research, University of Tasmania, 7000 Hobart, TAS Australia; 6 Discipline of General Practice, Adelaide Medical School, University of Adelaide, 5005 Adelaide, SA Australia; 7 IMPACT – the Institute for Mental and Physical Health and Clinical Translation, School of Medicine, Deakin University, Barwon Health, Geelong, Australia; 8 Orygen, The National Centre of Excellence in Youth Mental Health, Department of Psychiatry, Florey Institute for Neuroscience and Centre for Mental Health, University of Melbourne, Parkville, Victoria Australia

**Keywords:** Social Support, Social Isolation, Loneliness, Cardiovascular Diseases, Geriatrics, Aging, Interpersonal Relations

## Abstract

**Background:**

Poor social health is associated with increased risk of cardiovascular disease (CVD). Recent research suggests that different social health domains should be considered separately as the implications for health and possible interventions may differ.

**Aim:**

To assess social isolation, low social support and loneliness as predictors of CVD.

**Methods:**

Secondary analysis of 11,486 community-dwelling, Australians, aged 70 years and over, free of CVD, dementia, or significant physical disability, from the ASPirin in Reducing Events in the Elderly (ASPREE) trial. Social isolation, social support (Revised Lubben Social Network Scale), and loneliness were assessed as predictors of CVD using Cox proportional-hazard regression. CVD events included fatal CVD, heart failure hospitalization, myocardial infarction and stroke. Analyses were adjusted for established CVD risk factors.

**Results:**

Individuals with poor social health were 42 % more likely to develop CVD (p = 0.01) and twice as likely to die from CVD (p = 0.02) over a median 4.5 years follow-up. Interaction effects indicated that poorer social health more strongly predicted CVD in smokers (HR 4.83, p = 0.001, p-interaction = 0.01), major city dwellers (HR 1.94, p < 0.001, p-interaction=0.03), and younger older adults (70-75 years; HR 2.12, p < 0.001, p-interaction = 0.01). Social isolation (HR 1.66, p = 0.04) and low social support (HR 2.05, p = 0.002), but not loneliness (HR 1.4, p = 0.1), predicted incident CVD. All measures of poor social health predicted ischemic stroke (HR 1.73 to 3.16).

**Conclusions:**

Among healthy older adults, social isolation and low social support may be more important than loneliness as cardiovascular risk factors. Social health domains should be considered in future CVD risk prediction models.

**Supplementary Information:**

The online version contains supplementary material available at 10.1186/s12877-021-02602-2.

## Introduction

Cardiovascular disease (CVD) is the leading cause of morbidity and mortality worldwide [1, 2] and carries a high economic burden [3]. To reduce the significant health and economic burden associated with CVD, prevention can be improved by identifying and intervening upon factors that increase the risk of CVD. Poor social health is one possible factor that warrants further exploration. In 2016, a systematic review of 23 studies concluded that individuals with poor social health were 30 % more likely to experience coronary heart disease (CHD) and stroke events [4]. Social health refers to an individual’s ability to form satisfying and meaningful relationships, their ability to adapt in social situations, and their interactions with and perceived support from other people, institutions and services. Social health is often conceptualised into the constructs of social isolation, social support and loneliness. Social isolation is an objective measure of the lack of social relationships or infrequent social contact with others, while social support is a subjective measure of the actual or perceived availability of resources from others, and loneliness is a subjective negative feeling of being isolated [5]. Poor social health also imposes a large economic burden. In Australia, the estimated economic cost of loneliness is AUD$1.7 billion through absenteeism, caring, lost productivity and employee turnover [6]. However, this estimate does not take into account the additional burden from poor social health on the health care system through more general practitioner visits, medication use, accident and emergency service use, outpatient appointments, hospital stays, and nursing home admissions [7, 8]. Part of the high healthcare use is due to the increased health consequences associated with loneliness, however part of it is due to lonely people being *“more likely to seek medical assistance to satisfy their need for interaction and interpersonal stimulation”* (even after accounting for their physical health, age, gender, and socioeconomic status) [9].

There are a number of conceptual frameworks illustrating the underlying associations between social health and health [10–16]. As described by Ong et al. [16], the health impacts of these underlying associations with social health may be most apparent in later life. The broad pathway tends to be from social health; through socio-demographics, the sociological environment and chronic disease risk-factors; leading to chronic mental and physical ill-health and mortality. Particularly relevant to our study, Xia & Li [15] detail the molecular mechanisms along the pathway from social isolation and loneliness to CVD. Howick et al. [11] provides evidence that the direction from strong and supportive social relationships is a causal factor for better health and longevity. However, each component is often linked with bi-directional arrows indicating that the pathway is not clear, “*with health and social relationships interacting to influence each other, in virtuous circles or spirals of despair”* [11]. The bi-directional arrows are also present in Hodgson et al.’s [10] conceptual framework of the mechanisms linking social health to cardiovascular disease, which is specifically relevant to our study. The bi-directional arrows in the conceptual frameworks account for the health selection model, which explains how deterioration in health (such as a CVD event or decline in cognitive functioning) may limit or reduce social involvement. It is also important to understand the upstream determinants of poor social health such as personality [17]. Maladaptive premorbid personality is associated with difficult interpersonal relationships and also impedes adaptive health behaviour through adverse lifestyle habits and higher rates of non-adherence to medication. Personality disorder is thus linked to higher medical comorbidity.

Historically, the social health domains of social isolation, lack of social support and loneliness have been conflated or measured conjointly. However more recently, research has highlighted that social isolation, social support and loneliness need to be considered as distinct yet interconnected concepts [18] and assessed individually and simultaneously [19], as different social health constructs are likely to have different implications for health and well-being [5]. Due to the historical conflation of social health domains, there is a limited understanding of how these domains individually influence CVD risk. A recent synthesis of existing literature has provided a conceptual framework of the mechanisms linking loneliness and social isolation to cardiovascular disease, however, the authors stated that a direct comparison between the social health constructs were not possible due to limited published data [10].

Compared to other CVD risk factors such as elevated cholesterol or blood pressure, diabetes, significant family history, smoking, poor nutrition, physical inactivity, adiposity and depression, understanding of the link between social health and CVD is limited. To develop effective preventive interventions and guide cost-effective policy, a clear understanding of the extent to which social isolation, social support, and loneliness each influence CVD is required and how social health measures interact is important for identifying the most vulnerable populations for intervention.

The risk of poor social health becomes greater as we age, with a steep rise in poor social health among those aged 80 years or more [7, 20, 21]. Older age can be seen as a time to enjoy life and undertake activities that have been put off due to other pressures. However, the reality is that it can also be a challenging stage of life with the occurrence of negative life events and adjustment to life change. While some people choose to retire, others may enter retirement due to redundancy and encounter financial strain earlier than expected. It may be a time that calls for downsizing or moving house. Increased responsibility, such as care giving or financial planning, may be tiring. Death of a partner, relative or close friend, whether it is a sudden event or is preceded by prolonged disability, can have devastating emotional and/or financial consequences. Loss of independence, such as disqualification of a driving license, may be confronting and limit the availability of convenient transport. Disability arising from age-related health conditions, including cognitive decline, affects the opportunity to engage socially and can trigger loneliness.

We aimed to assess social isolation, low social support and loneliness as predictors of incident CVD, in addition to established risk factors assessed by current CVD risk prediction models. We utilised a large contemporary cohort of healthy, community-dwelling Australians aged 70 years and over who were free of CVD at baseline, who were followed for an average of five years. Having a healthy sample reduces the likelihood of reverse causality, where a prior CVD, other chronic disease or preceeding symptoms could lead to poor social health [22]. Notably, this is the first assessment of loneliness as a predictor of CVD incidence among adults aged 75+ years.

## Methods

### Study sample

This is a secondary analysis utilising data from the ASPirin in Reducing Events in the Elderly (ASPREE) study, and the ASPREE Longitudinal Study of Older Persons (ALSOP) questionnaire sub-study. Ethics approval was received from the Monash University Human Research Ethics Committee, and all participants provided written informed consent. During 2010-2014, 19,114 community-dwelling healthy older adults with no overt disease likely to cause death in the next five years were recruited [23]. All participants provided written informed consent at recruitment. Exclusion criteria included prior CVD events, presence of function limiting physical disability, or major cognitive impairment [23]. Follow-up was completed on 12 June 2017. Low-dose aspirin was found to have no significant effect on the primary end-point of disability free survival, nor cardiovascular disease events, over a median follow-up of 4.7 years [24, 25]. In ASPREE, 1.55 % (*n *= 296) of participants were lost to follow-up and 1.24 % (*n *= 237) withdrew consent during this time [24].

Eighty-nine percent (*n *= 14,892) of Australian ASPREE participants also participated in ALSOP, and most (>85 %) within 15 months of enrolling in ASPREE [26]. Most (87 %) completed both the ALSOP medical and social questionnaires. Participants were excluded from this analysis if they had incomplete social health data (*n *= 1,367) or reported living in residential care facilities or nursing homes at the time of the first ALSOP questionnaire completion (*n* = 31). Excluded participants were more likely to be older, women, have more education, and have better high-density lipoprotein cholesterol (+0.04 mmol/L, *p* = 0.004), Table 1. However, there were no differences between excluded and included participants in terms of social health, ethnicity, residential region, smoking, the number of CVD risk factors, systolic blood pressure (*p *= 0.8), non-HDL (*p* = 0.7), diabetes (*p* = 0.08), serum creatinine (*p* = 0.2), antihypertensive drug use (*p* = 0.4), CVD incidence (*p* = 0.4), or CVD mortality (*p* = 0.4).

**Table 1:** Baseline characteristics of included and excluded participants

		Included ( *n* = 11,486)		Excluded (*n* = 1,398)		p–value
		**n**	**%**	**n**	**%**	
**Age, years**						
	mean±SD	75.03±4.22		76.80±4.82		<0.001
	70 < 75	6,944	60 %	602	43 %	<0.001
	75 < 80	2,950	26 %	435	31 %	
	≥ 80	1,592	14 %	361	26 %	
**Gender**						
	Female	6,126	53 %	880	63 %	<0.001
	Male	5,360	47 %	518	37 %	
**Social Isolation**						
	No	11,262	98 %	n < 5 in a cell		0.6
	Isolated	224	2 %			
**Social Support**						
	High	11,258	98 %	1,067	98 %	0.6
	Low	228	2 %	19	2 %	
**Loneliness**						
	No	10,927	95 %	1,313	94 %	0.1
	Lonely	559	5 %	82	6 %	
**Composite Social Health** ^a^						
	Positive	10,576	92 %	n < 5 in a cell		0.3
	Poor	910	8 %			
**Ethnicity**						
	White/Caucasian	11,345	99 %	1,372	98 %	0.09
	Not	136	1 %	24	2 %	
**Education**						
	≤12 years	6,701	58 %	915	65 %	<0.001
	>12 years	4,785	42 %	483	35 %	
**Region**						
	Major city	6,112	53 %	719	52 %	0.2
	Inner regional	4,060	35 %	496	36 %	
	Outer regional/remote	1,289	11 %	176	13 %	
**Smoking Status**						
	Current	328	3 %	30	2 %	0.1
	Former	4,754	41 %	553	40 %	
	Never	6,404	56 %	815	58 %	
**Number of CVD risk factors** ^b^						
	mean±SD	2.00±1.03		2.03±1.00		0.3
	0–1	3,713	32 %	448	32 %	0.2
	2	3,399	30 %	388	28 %	
	3–5	4,361	38 %	560	40 %	

^a^ The social health composite categories were defined as positive (not isolated, supported, and not lonely), or poor (isolated, not supported and/or lonely)

^b^ Number of five CVD risk factors (current tobacco smoking, hypertension, antihypertensive drug use, dyslipidemia, diabetes)

### Social health

As we were interested in assessing whether social health could be incorporated into CVD risk models, the social health questions needed to be readily interpretable. There are no established or validated cut-offs for social health measures. However, the majority of studies in Valtorta et al. [4].’s relevant systematic review have created continuous scores based on several questions, with some comparing the highest versus lowest categories. This approach has helped determine a link between poor social health and CVD, despite the exact social health measures differing in the various studies. However, this approach limits translation to public health messaging as the actual level of social isolation, social support or loneliness is difficult to determine, and there is not a universally agreed method for assessment of social health. For example, it is difficult to determine what the lowest or highest categories represent in terms of number of close friends, social contacts and community activities. Clear public health messages akin to having a systolic blood pressure less than 120 mm/Hg would be of benefit for research translation. Furthermore, we hypothesise that there is likely a threshold of optimal social health for CVD benefit and that the relationship is not linear. Hence, we have assessed social health as dichotomous categories that supports application to broader settings. In our study, social isolation was defined as engaging in community activities less than once per month and having contact with four or fewer relatives and close friends in a month. Social support was defined as having four or more relatives or close friends with whom private matters could be discussed, or be called upon for help. From the validated Revised Lubben Social Network Scale [27] (LSNS) collected through ALSOP, we utilised two questions that pertained to social isolation: *“How many of your friends/relatives do you see or hear from at least once a month?”* with six response options (*none, one, two, three-four, five-eight, nine or more*) and *“How often do you: a) Go to a club, local organisation, neighbour-hood or other small group? b) Attend an educational class? c) Go to a church, temple or other place of worship, or take part in related activities?“* with five response options (*never, rarely – less than once a month, sometimes – 1-3 times a month, often – once a week or more, always – most days*) and four questions that pertained to social support: “*How many friends/relatives do you feel at ease with that you can talk about private matters?“* and *“How many friends/relatives do you feel close to such that you could call on them for help?“*, with six response options (as above). Loneliness was defined by feeling lonely occasionally (3-5 days/week) or all of the time (5-7 days/week) based on an item from the Center for Epidemiological Studies – Depression (CESD) Scale: “*During the past week I felt lonely*” which was collected as part of ASPREE. For sensitivity analyses, two approaches were employed to calculate social health measures as continuous. First, each response category was sequentially numbered. Second, the response categories were recoded as values, for example, “three-four” became 3.5. In the second scenario, “nine or more” was recoded as 9, “sometimes” as 2, “often” as 8, and “always” as 24. Social isolation scales ranged from 0-21 (scenario 1) and 0-85 (scenario 2), social support from 0-20 and 0-36, and loneliness from 0-3 and 0-6. The social health composite categories were defined using the binary categories of social health as positive (not isolated, supported, and not lonely), or poor (isolated, not supported and/or lonely).

### Cardiovascular disease

The main outcomes were incident CVD and fatal CVD, and subtypes are major adverse cardiovascular events (MACE), heart failure hospitalization, MI and stroke. Incident CVD was a prespecified secondary endpoint of ASPREE, adjudicated by an expert committee [24]. Participants were followed until either the data-cut date (12 June 2017), or when they had a CVD incident, or censored at the point where contact was lost (i.e. competing event or withdrawal). Full details have been provided previously  [24]. Incident CVD included fatal or nonfatal myocardial infarction (MI), heart failure hospitalization, and fatal or nonfatal stroke. MACE included incident CVD, but excluded heart failure and haemorrhage stroke. Nonfatal MI was defined according to the joint guidelines of the European Society of Cardiology and the American College of Cardiology [24]. Heart failure hospitalization was defined as any unplanned overnight stay or longer in a hospital or similar facility with heart failure as the principal reason for admission. The criteria for the diagnosis of nonfatal stroke were identified by the World Health Organization (WHO) [24].

### Potential confounders

As we were interested in assessing whether social health is a CVD risk factor beyond established CVD risk factors, we considered potential confounders to be those already incorporated in a primary CVD risk assessment tool developed specifically from this cohort [28]. As sensitivity analyses, additional models adjusting for socio-demographic, lifestyle and depressive symptoms covariates outlined by the Heart Foundation of Australia [29] were examined.

### Stratification

Current CVD risk assessment tools [30, 31] could be improved through incorporation of newly identified CVD risk-factors. As social health may be one such CVD risk-factor, stratification was undertaken to assess interaction effects. The analysis assessing composite social health as a predictor of CVD incidence was stratified by socio-demographics (age, gender, partner status, ethnicity, education, SEIFA [32], residential region) and CVD risk factors (smoking hypertension anti-hypertensive use, dyslipidaemia, diabetes).

### Sample size and statistical power

Based upon a Type I error of 0.050 (two-tailed), power of 80 %, an exposure rate of 8 % (to poor social health at the study baseline visit [33, 34], Under review) and a hazard ratio of 1.30^4^, 74 cardiovascular events would be needed to examine the main aim of whether social health predicts CVD. Among the 11,486 included participants, there were 487 nonfatal and 83 fatal CVD events over the median 4.7 years of follow-up.

### Statistical analysis

The correlation between social isolation, social support, and loneliness was assessed, and then the associations with CVD using Cox proportional hazards regression. Competing events for fatal CVD (cancer death, major haemorrhage death, other death) were also assessed. The main analysis was adjusted for covariates in a CVD risk assessment tool developed specifically from this cohort [28] and sensitivity analyses were undertaken with further adjust for socio-demographic, lifestyle and depressive symptoms covariates. To test robustness of the main aim, participants censored or with CVD in the first half-year, and then year, were excluded to account for potential for reverse causality and the delay between ASPREE baseline and ALSOP questionnaire completion. Finally, sensitivity analyses assessed social health as continuous measures. Analyses were performed using Stata version 15.1. A p-value of <0.05 was be used to determine statistical significance.

## Results

The final sub-cohort consisted of 11,486 (53 % women, mean age 75.03±4.22SD) community-dwelling older Australians, Table 1. The majority of participants had positive composite social health (92 %) at baseline, with only a few reporting isolation (2 %), low support (2 %) or loneliness (5 %). There was some crossover, as participants who were classified as being socially isolated were also much more likely to report low social support than participants not socially isolated (29 % versus 1 %, p < 0.001). Similarly, participants who were socially isolated or had low social support were more likely to report being lonely (Social isolation: 10 % vs. 5 %, p = 0.001; Low social support: 9 % vs. 5 %, p < 0.001). While 8 % were categorised as having poor social health (either being socially isolated, having low social support or being lonely), only 0.05 % (n = 6) were categorized as being poor on all three (reported being socially isolated, having low social support and being lonely).

Prior work has demonstrated that social isolation, social support and loneliness displayed diverse relationships with CVD risk factors and risk scores in this cohort [34]. Physical inactivity and experiencing depressive symptoms were the only consistent CVD risk factors associated with all three social health domains [34]. This prior work emphases the importance of distinguishing between these three domains.

There were 487 (4.2 %) first time CVD events during the 50,887 person-years of observation (mean±SD follow-up of 4.43 ± 1.3 years, median 4.51, Interquartile Range (IQR) 3.48-5.53, range 0–7). First time CVD events occurred on average at age 80.1 ± 5.6 SD years (median: 79.1, IQR 75.6-84.0, range 70.6-96.4). There were 83 (0.7 %) CVD deaths during the 52,353 person-years of observation (mean ± SD 4.55 ± 1.2 years, median 4.61, IQR 3.58-5.60, range 0–7). CVD deaths occurred on average at age 82.9 ± 6.3SD years (median: 82.9, IQR 76.9-87.6, range 72.1-94.7).

### Social health as a risk factor for cardiovascular disease

**Fig. 1:**
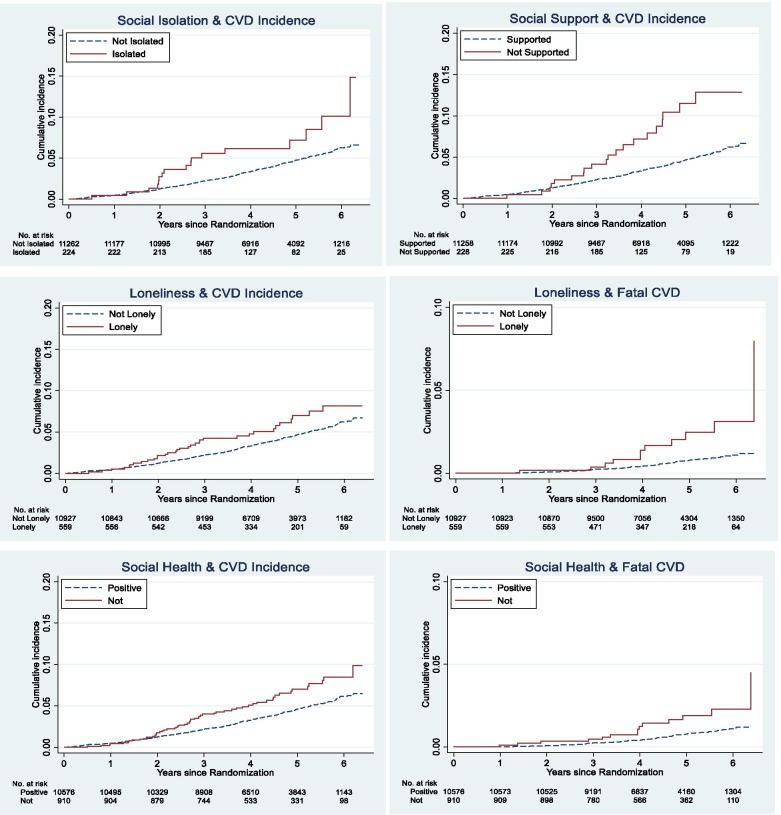
Cumulative incidence and mortality of cardiovascular disease by baseline social health status, *n* = 11,486

Poor social health as a composite measure predicted both incident CVD and fatal CVD (Fig. 1 & Additional file 1: Appendix 1 age-adjusted model). Individually, social isolation and low social support (but not loneliness) predicted incident CVD events. There were too few fatal CVD events among participants who were isolated or had low support to assess the relationship, however, we were able to assess loneliness, which predicted fatal CVD. After adjusting for traditional risk factors (Table 2), these relationships remained with a slightly lower magnitude of association (≤10 %).

**Table 2:** Social health as a predictor of incident and fatal cardiovascular disease^a^ over 5 years for older adults recruited between 2010 and 2014 in Australia, *n* = 11,486

**End point**	**Social isolation**								**Social support**							
	**Not ** **(*n* = 11,262)**		**Isolated ** **(*n*= 224)**		*Hazard Ratio*^*h*^	*p–value*	*95 % Confidence Interval*		**High ** **(*n* = 11,258)**		**Low ** **(*n* = 228)**		*Hazard Ratio*^*h*^	*p–value*	*95 % Confidence Interval*	
	*events*	*rate*	*events*	*rate*					*events*	*rate*	*events*	*rate*				
Cardiovascular disease^b^	470	41.7	17	75.9	**1.66**	**0.04**	1.02	2.70	467	41.5	20	87.7	**2.05**	**0.002**	1.31	3.21
Fatal cardiovascular disease^c^	82	7.3	1	4.5	*n < 5*				81	7.2	2	8.8	*n < 5*			
*Major adverse cardiovascular event*^*d*^	360	32.0	10	44.6	1.21	0.6	0.64	2.27	356	31.6	14	61.4	**1.79**	**0.03**	1.05	3.07
*Hozpitalization for heart failure*^*e*^	94	8.3	5	22.3	**2.58**	**0.04**	1.04	6.39	96	8.5	3	13.2	*n < 5*			
*Fatal or nonfatal myocardial infarction*^*f*^	201	17.8	6	26.8	1.24	0.6	0.55	2.81	202	17.9	5	21.9	1.09	0.9	0.45	2.65
*Fatal or nonfatal ischemic stroke*^*g*^	189	16.8	9	40.2	**2.34**	**0.01**	1.19	4.59	186	16.5	12	52.6	**3.16**	**<0.001**	1.76	5.68
**End point**	**Loneliness**								**Social Health composite**^**i**^							
	**Not ** **(*n* = 10,927)**		**Lonely ** **(*n* = 559)**		*Hazard Ratio*^*h*^	*p–value*	*95 % Confidence Interval*		**Poor ** **(*n* = 910)**		**Positive ** **(*n* = 10,576)**		*Hazard Ratio*^*h*^	*p–value*	*95 % Confidence Interval*	
	*events*	*rate*	*events*	*rate*					*events*	*rate*	*events*	*rate*				
Cardiovascular disease^b^	454	41.5	33	59.0	1.4	0.10	0.95	1.93	56	61.5	431	40.8	**1.42**	**0.01**	1.07	1.88
Fatal cardiovascular disease^c^	72	6.6	11	19.7	**2.55**	**0.004**	1.34	4.83	14	15.4	69	6.5	**2.00**	**0.02**	1.12	3.60
*Major adverse cardiovascular event*^*d*^	343	31.4	27	48.3	**1.50**	**0.045**	1.01	2.22	41	45.1	329	31.1	1.35	0.07	0.97	1.87
*Hozpitalization for heart failure*^*e*^	94	8.6	5	8.9	0.87	0.8	0.35	2.15	87	8.2	12	13.2	1.41	0.3	0.77	2.59
*Fatal or nonfatal myocardial infarction*^*f*^	194	17.8	13	23.3	1.34	0.3	0.76	2.36	186	17.6	21	23.1	1.24	0.4	0.79	1.95
*Fatal or nonfatal ischemic stroke*^*g*^	181	16.6	17	30.4	**1.73**	**0.03**	1.05	2.86	171	16.2	27	29.7	**1.76**	**0.007**	1.17	2.65

^a^ As some end points were composites, a participant who had events for more than one component of the composite (e.g., stroke and then acute myocardial infarction) would contribute only the first event that occurred to the composite end point but would contribute an event to the separate analyses of each component. Hence, summation of the number of events for separate components of a composite end point does not equate to the number of events for the composite end point. If there are fewer than five participants in a cell, then statistics are not reported to preserve participant’s privacy and potential unreliable statistical inferences

^b^ CVD incidence, a prespecified secondary end point, was a composite of fatal CHD (death from myocardial infarction, sudden cardiac death, or any other death in which the underlying cause was considered to be CHD), nonfatal myocardial infarction, fatal or nonfatal stroke (including haemorrhagic stroke), or hospitalization for heart failure. 50,887 person-years of observation (mean 4.43 ± 1.28SD years; median 4.51, IQR 3.48-5.53, range 0–7)

^c^ Fatal CVD was defined as any death from stroke (including haemorrhagic stroke) or CHD. 52,353 person-years of observation (mean 4.55 ± 1.21SD years, median 4.61, IQR 3.58-5.60, range 0–7). Fatal CVD assessed for competing events (cancer death, major haemorrhage death, other death)

^d^ Major adverse cardiovascular events, a non-prespecified end point, was a composite of fatal CHD (excluding death from heart failure), nonfatal myocardial infarction, or fatal or nonfatal ischemic stroke. 51,063 person-years of observation (mean 4.44 ± 1.26SD years, median 4.52, IQR 3.48-5.54, range 0–7)

^e^ 51,497 person-years of observation (mean 4.48 ± 1.24SD years, median 4.54, IQR 3.51-5.56, range 0–7)

^f^ 51,297 person-years of observation (mean 4.47 ± 1.25SD years, median 4.54, IQR 3.50-5.55, range 0–7)

^g^ Data for ischemic stroke included cases that were adjudicated as ischemic stroke, cases for which stroke type was uncertain after adjudication, and cases of ischemic stroke with haemorrhagic transformation. 51,349 person-years of observation (mean 4.47 ± 1.25SD years, median 4.54, IQR 3.50-5.55, range 0–7)

^h^ Adjusted based on a primary CVD risk assessment tool developed specifically from this cohort 28: age (years), gender (women, men), smoking (never, past, current), systolic blood pressure (mmhg), high-density lipoprotein (HDL-c; mmol/L), non-HDL (mmol/L), diabetes (yes, no), serum creatinine (mg/dL), and antihypertensive drug use (yes, no)

^i^ The social health composite categories were defined as positive (not isolated, supported, and not lonely), or poor (isolated, not supported and/or lonely)

In sensitivity analyses further adjusting for socio-demographic, lifestyle and depressive symptoms CVD risk factors, these relationships remained with, again, a lower magnitude of association and a few lost statistical significance (Additional file 1: Appendix 1). When inclusion was restricted to participants still enrolled and without events after six-months or one-year from ASPREE baseline, the magnitude of associations became stronger (Additional file 1: Appendix 2).

### Social health as a risk factor for cardiovascular disease subtypes

Social isolation, low support, loneliness and poor composite social health consistently predicted ischemic stroke events (Table 2). In sensitivity analyses adjusting for socio-demographic and lifestyle CVD risk factors, the associations between poor social health and stroke held except for loneliness, which was attenuated and lost statistical significance when adjusted for depressive symptoms (Additional file 1: Appendix 1). No other distinguishable pattern among CVD subtypes emerged; social isolation predicted heart failure hospitalization, while low social support and loneliness predicted MACE (Table 2).

### Subgroup analysis of social health as a predictor of cardiovascular disease

**Fig. 2:**
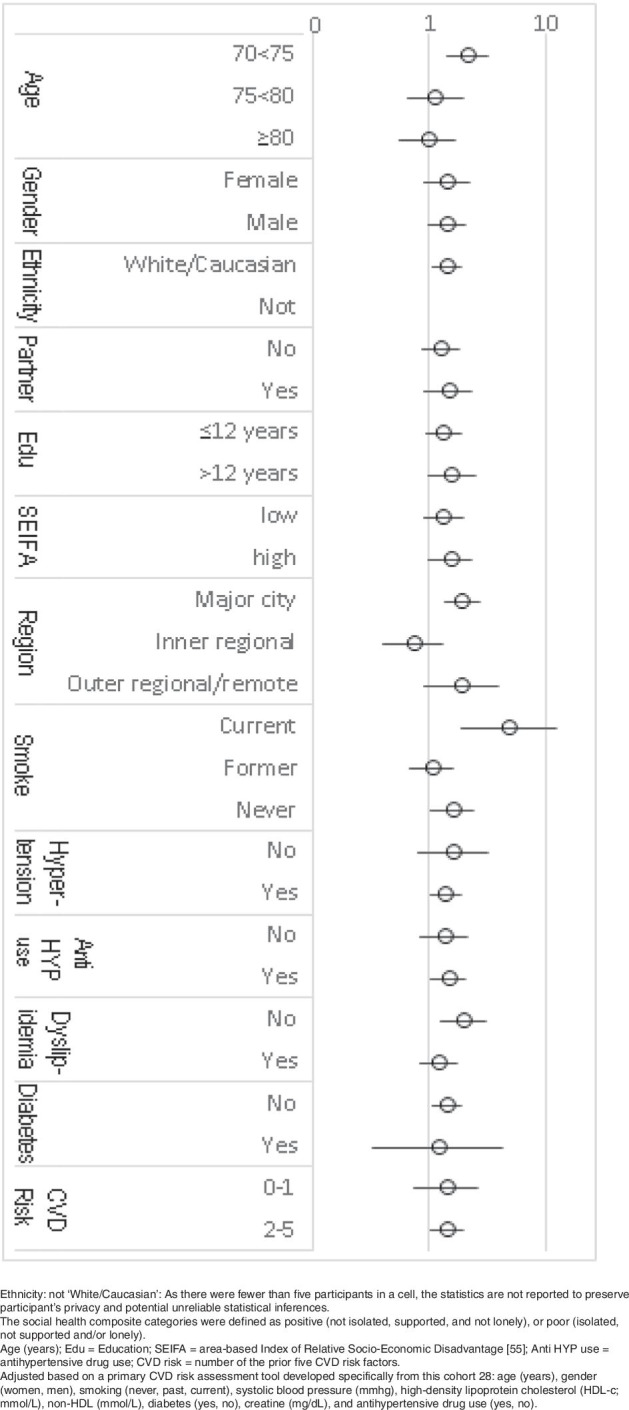
Subgroup stratification: Social health as a predictor of incident cardiovascular disease over five years for older adults recruited between 2010 and 2014 in Australia, *n* = 11,486

Age, smoking and residential region modified the association between social health and CVD risk (p-interaction: 0.01, 0.01, 0.03 respectively), Fig. 2. Poor social health increased the risk of CVD almost 5–fold among smokers (HR 4.83, *p* = 0.001), and doubled the risk among participants aged 70-75 years (HR 2.12, *p* < 0.001) or participants living in a major city (HR 1.94, *p* < 0.001).

### Alternative assessment of social health measures

When social health measures were assessed on continuous scales, no strong patterns with CVD outcomes were observed (Additional file 1: Appendix 3).

## Discussion

Individuals with poor social health were 42 % more likely to develop CVD and twice as likely to die from CVD over a five year period among community-dwelling, older adults, who were free of diagnosed CVD and dementia at baseline. Poor composite social health more strongly predicted incident CVD among participants who were currently smoking, living in a major city, or aged 70 to 75 years. In regards to the individual components of social health, there were too few fatal CVD events among participants who were isolated or had low support to assess the relationship. However, social isolation and low social support predicted incident CVD, and loneliness predicted fatal CVD. In regards to the CVD subtypes, all measures of poor social health consistently predicted ischemic stroke. Additionally, isolation predicted heart failure hospitalization, while low support and loneliness predicted MACE.

### Social health as a risk factor for cardiovascular disease

The magnitude of association reported here between poor social health and incident CVD aligns with a systematic review of 23 studies from 16 datasets with 4,628 CHD and 3,002 stroke events over 3 to 21 years [4]. However, that review did not investigate which components of social health were driving these associations. We show that the risk of incident CVD increased by 66 % if individuals were socially isolated and doubled if individuals had low social support, however no association was observed with loneliness. There have been very few prior studies which have investigated the association between loneliness and CVD incidence; with three [35–39] reporting an association and one [36] reporting no association.

However, there are a few discrepancies between these prior studies which make it difficult to compare our findings; two [35, 37] observed the association among women, one [37] was restricted to daytime loneliness for women homemakers, in two [36, 38, 39] loneliness measures incorporated aspects of social isolation and/or social support, and the CVD measures varied between studies. In detail; among 353 American women homemakers from the Framingham study, feeling lonely during the day (one-item) was associated with MI and coronary death over twenty years (HR 4.0, *p* = 0.03) [37]. Among 2,616 Americans aged 25-74 years from the National Health and Nutrition Examination Survey, the same single loneliness question as used in our study (CESD) was associated with CHD incidence over 15 years among women (low vs. high: HR 1.81, *p* < 0.001), but not men [35]. Among ~5,000 British aged 50+ years from the English Longitudinal Study of Ageing (ELSA), loneliness (assessed as lack of companionship, isolation, and being left out) was associated with heart disease and stroke over 5.4 years [39] (OR 1.27, *p* < 0.001) and CVD incidence over 9.6 years [38] (low vs. high: self-reported CVD HR 1.30, p < 0.001; CVD-related hospital admissions HR 1.48, *p* < 0.001). Among 479,054 British aged 40–69 years from the UK Biobank study, loneliness (assessed as feeling lonely and unable to confide in someone close) was not associated with acute MI and stroke incidence over 7.1 years. [36] Among these four cohorts, two also assessed social isolation and reported no association with heart disease and stroke [39], MI and coronary death [37], or CVD incidence [38].

Notably we are the first to assess loneliness as a predictor of CVD incidence among adults aged 75+ years, therefore our findings may indicate that social isolation and social support are more important than loneliness for cardiovascular longevity in later life. Additionally, we are the first to assess all three social health constructs separately, and our findings highlight the importance of considering aspects of social health beyond perceived loneliness.

### Subgroup analysis of social health as a predictor of cardiovascular disease

Three subgroups had a greater risk of incident CVD from poor social health, indicating interaction effects. Given the magnitudes of these interaction effects, the combination of poor social health with smoking, residential location and/or age is particularly important as a possible inclusion in future CVD prediction tools.

First, we identified that among individuals who smoke, poor social health increased the risk of CVD almost 5–fold compared to smokers with good social health. Smoking is a well-established, modifiable risk factor for non-communicable diseases, including CVD, and mental health disorders like depression. The benefits of quitting have been communicated through decades of public health campaigns and tobacco control policies. However, this has created a negative smoking stigma, especially among vulnerable groups who find it difficult to quit [40]. Smoking stigma incorporates aspects of shame, social isolation, and discrimination, and may compound stigma experiences in other areas [40]. Additionally, Australian tobacco control policies prohibit smoking inside public spaces, and have contributed to the general decline in smoking rates [41], reducing the availability of peers with whom to share ‘social smoking’. Older adults would particularly have fewer opportunities for ‘social smoking’ as the rate of daily smokers is particularly low [41], partly likely due to smoking being such a strong risk factor for life-limiting disease and death.

Second, among individuals living in a major city, poor social health doubled the risk of incident CVD. International and national research has suggested that people who live in rural, outer metropolitan fringe or lower socio-economic locations are at greater risk of social isolation and loneliness [7, 20, 42]. However among older Australians, contradictory to these findings, “*social isolation is more prevalent in both the largest urban [city] centres and in the most substantial, and sparsely populated, territories” *[43, 44]. Hence, the interaction effect of living in a major city and poor social health with increased incident CVD may be specific to older adults. Potentially, a greater sense of community in regional areas, compared to Australian cities, may be the cushioning benefit [45]. Pretty and colleagues [45] suggest that *“beyond social support (itself a major positive factor for many with health issues) the sense of community provides a buffer against physical and psychological symptoms of illness, and facilitates adjustment.”* For example, a sense of community may be particularly important for older adults with children who have moved away or family and friends who have passed away or moved into care.

Third, among individuals aged 70-75 years, poor social health doubled the risk of CVD. The risk of poor social health becomes greater as we age due to the occurrence of life events including retirement, financial strain, downsizing, poorer health, disability, cognitive decline, loss of independence, moving into care and bereavement [7, 20, 21].

### Social health as a risk factor for of cardiovascular disease subtypes

The link between poor social health and stroke is consistent with the systematic review of 23 studies by Valtorta et al. [4]. However, our magnitude of association for composite social health as a predictor of stroke was higher than the previous overall estimate (76 % vs. 32 % [4]). Notably the previous systematic review predominantly assessed social isolation (*n* = 18/23 included studies) but included measures of low social support (*n* = 1) and loneliness (*n* = 3). Our magnitude of association for social isolation as a predictor of ischemic stroke was even higher (216 %). Potentially the difference in the magnitude of association could be a reflection of the greater age of our cohort (≥70 years), compared to those encompassed by systematic review (all ages). We also add that each separate social health component was associated with incident stroke, and low social support having the strongest effect. Other associations were less consistent across CVD subtypes and social health components; specifically, social isolation predicted heart failure hospitalization, and low social support and loneliness predicted MACE. However, these less consistent association could be driven, at least in part, but low power for some of the analyses.

### Strengths & limitations

When interpreting our finding it is important to note that our aim was to assess the contribution of social health as a predictor of incident CVD, beyond current CVD risk prediction models. Hence, analyses were adjusted for CVD risk factors in an established prediction model. As these CVD risk factors are on the causal pathway between social health and CVD, such adjustment likely leads to an underestimate of the importance of social health for cardiovascular health. However, our minimally adjusted models were fortunately only slightly (<10 %) stronger in magnitude when compared our main analysis adjusting for a CVD risk prediction tool. Furthermore, our assessment of social health was based on potential questions that could be incorporated into CVD risk models. Our continuous assessment of social health illustrates that the association with CVD may not be linear, and that there is likely a threshold for optimal social health. Furthermore, prior social health assessment based on continuous scores would be difficult to incorporate into a CVD model. Given that our cohort were healthy and had good social health, our continuous score findings are likely not comparable to prior studies.The main limitation of this study is that our sample had an expectedly lower prevalence of poor social health compared with prior population estimates [6, 7, 46], and coupled with the five year follow-up period there were not enough fatal CVD events to assess social isolation or social support as predictors. Furthermore, the sensitivitiy analysis expanding loneliness to three categories reduced the number of poor social health cases in the reference category, and likely reduced the power for statistical inference. In such a relatively healthy cohort, a longer follow-up period would be optimal. Furthermore, assessment of social health over a longer period of time would provide the opportunity to assess the contribution of longitudinal changes in social isolation, social support, and loneliness, including persistent (severe, long-term) poor social health. However, the healthy sample is also a strength as it reduces the likelihood of reverse causality, where an incident CVD or preceeding symptoms could lead to reduced social health [22] Furthermore, each generation of older people have comparatively greater mental and physical capabilities and this older healthy cohort is likely representative of future generations [47]. People may feel embarrassed or uncomfortable acknowledging that they are experiencing poor social health, particularly given that there is a potential harmful stigma to being labelled as “lonely” by a health care provider [5]. However, under-reporting of poor social health would result in our effect estimates being conservative. Inconsistency in social health measures is a common limitation of this research area [48, 49] and we acknowledge that dichotomisation, undertaken to compare to prior findings and assess social health as a composite, may not be optimal. However, a single-item measure of loneliness is commonly used, has been acknowledged as valid and is likely more appropriate for an older age group [50]. Loneliness was part of the depressive symptoms scale, however there was no difference if adjustment included or excluded the loneliness item in the depressive symptoms score (data not shown). Heart failure hospitalization may have been influenced by poor social health [8], which may have contributed to the stronger association with social isolation. As participants were relatively healthy, mainly white and community-dwelling, generalizability may be restricted due to culture, healthcare systems, and socio-economic standing [51]. Additional common limitations of cohort studies include the healthy cohort effect and the fact that participation may influence the variable of interest (in this case social health).

Strengths of this study include analysis of a large, well-characterized population-based cohort of older adults with a very high response rate to our survey instrument. Data had high integrity, very little loss to follow-up, validated and adjudicated measurement of the outcome (CVD), and low misclassification bias due to continuing review of medical records, even in the event of attrition. We are the first to identify an association between social health and CVD in an Australian sample [52], which is likely due to our validated, medically diagnosed measure of CVD (rather than self-report). It is known that socially isolated older adults are hard to recruit for research [53] and individuals with poor social health have more general practitioner visits [48, 54, 55], hence, a strength of this study is that recruitment was through general practice. Findings are generalizable to community-dwelling people who reach age 70 without overt CVD, dementia or other known life-limiting disease.

### Clinical implication

The aging population presents the challenge of supporting older people to maintain a healthy, fulfilling, independent and community-dwelling life for longer. Traditional CVD risk assessment tools [30, 31] concentrate on physical health. The incorporation of newly identified CVD risk factors (i.e. socio-demographics, lifestyle, mental health and social health) need to be explored to improve CVD risk prediction. In sensitivity analyses, we demonstrate that poor social health predicted fatal CVD and the relationship with incident CVD was only slightly attenuated with extensive adjustment for traditional, socio-demographic, lifestyle and depressive symptoms CVD risk factors. Further, poor social health (including each component) consistently predicted ischemic stroke regardless of adjusted covariates. Our findings that poor social health predicts incident CVD, fatal CVD and stroke, and the strong interaction effect with smoking, present a solid foundation to incorporate social health in future CVD risk prediction models. In the interim, health professionals are part of a multidisciplinary network and could identify patients who have poor social health for community supports. Even if health professionals cannot change their patients’ social circumstances, they could concentrate more on these high-risk individual’s CVD risk factors such as smoking, blood pressure and cholesterol.

## Conclusions

We observed that healthy, community-dwelling, older adults with poor social health were 42 % more likely to develop CVD and twice as likely to die from CVD over five years. Internationally, 6-10 % of older adults have poor social health [7, 20] and given the rapid growth in the number of older adults, the number of people affected will be substantial over the next decades. Here we present the first assessment of social isolation, social support and loneliness separately as predictors of incident CVD events. Our findings demonstrate that among healthy older adults, social isolation and low social support may be more important than perceived loneliness for cardiovascular health in later life.

Our findings highlight that poor social health predicts incident CVD events beyond biological CVD risk factors, and thus should be considered in future risk prediction models. Until social health is formally introduced into CVD prediction models, health professionals could utilise this information to identify individuals at high risk and intervene on their other CVD risk factors (such as smoking, blood pressure and cholesterol). Further, our findings inform future intervention work and policy shaping how a better understanding of social isolation, social support and loneliness can be built into our current CVD prevention and management practices to enhance their effectiveness.

## Supplementary Information

**Additional file 1.**

## Data Availability

The datasets generated and/or analysed during the current study are not publicly available due data being part of a large ongoing observational cohort study with a rigorous process to access data. The datasets generated and/or analysed during the current study are available in the ASPREE clinical trial data resource repository, https://aspree.org/aus/researchers/. The ASPREE clinical trial data resource is managed in partnership with the US, in the Australian ASPREE National Coordinating Centre. New ASPREE projects with appropriate scientific merit may be proposed by external researchers, and submitted to ASPREE for consideration. Project proposals requesting access to any aspect of data, samples, or analyses from the ASPREE clinical trial and/or sub-studies must gain the support of the ASPREE Principal Investigators. Applications are submitted via a secure website, the ASPREE Access Management System (AMS). Applicants can obtain information by contacting aspree.ams@monash.edu.
