# Supplementary material for: Social isolation, social support and loneliness as predictors of cardiovascular disease incidence and mortality
Source: BMC Geriatr. 2021 Dec 13;21:711. doi: 10.1186/s12877-021-02602-2 (PMC8684069; doi:10.1186/s12877-021-02602-2)
Supplement: Supplementary file 1 — Additional file 1. [file 12877_2021_2602_MOESM1_ESM.docx]

# Appendix 1: Sensitivity Analysis: Socio-demographic, life-style and depressive symptom adjustment: Social health as a predictor of incident and fatal cardiovascular disease^a^, n=11,486

| **Social isolation** |  | **Model 1: Age** | | | | **Model 2^h^** | | | | **Model 3^i^: 2 + Depressive Symptoms** | | | |
| --- | --- | --- | --- | --- | --- | --- | --- | --- | --- | --- | --- | --- | --- |
|  | *events* | *HR* | *p-value* | *95% CI* | | *HR^h^* | *p-value* | *95% CI* | | *HR^i^* | *p-value* | *95% CI* | |
| Cardiovascular disease^b^ | 487 | **1.88** | **0.01** | 1.16 | 3.04 | 1.43 | 0.2 | 0.86 | 2.37 | 1.41 | 0.2 | 0.85 | 2.34 |
| Fatal cardiovascular disease^c^ | 83 | *n<5* | | | | *n<5* | | | | *n<5* | | | |
| *Major adverse cardiovascular event^d^* | 370 | 1.42 | 0.3 | 0.76 | 2.66 | 1.10 | 0.8 | 0.58 | 2.07 | 1.08 | 0.8 | 0.57 | 2.04 |
| *Hozpitalization for heart failure^e^* | 99 | **2.85** | **0.02** | 1.16 | 7.01 | 2.05 | 0.2 | 0.74 | 5.67 | 2.01 | 0.2 | 0.72 | 5.59 |
| *Fatal or nonfatal myocardial infarction^f^* | 207 | 1.51 | 0.3 | 0.67 | 3.40 | 1.10 | 0.8 | 0.48 | 2.49 | 1.10 | 0.8 | 0.49 | 2.50 |
| *Fatal or nonfatal ischemic stroke^g^* | 198 | **2.47** | **0.01** | 1.26 | 4.81 | **2.16** | **0.03** | 1.09 | 4.26 | **2.09** | **0.03** | 1.06 | 4.14 |
| **Social support** |  | **Model 1: Age** | | | | **Model 2^h^** | | | | **Model 3^i^: 2 + Depressive Symptoms** | | | |
|  | *events* | *HR* | *p-value* | *95% CI* | | *HR^h^* | *p-value* | *95% CI* | | *HR^i^* | *p-value* | *95% CI* | |
| Cardiovascular disease^b^ | 487 | **2.15** | **0.001** | 1.37 | 3.36 | **1.81** | **0.01** | 1.14 | 2.88 | **1.79** | **0.01** | 1.13 | 2.85 |
| Fatal cardiovascular disease^c^ | 83 | *n<5* | | | | *n<5* | | | | *n<5* | | | |
| *Major adverse cardiovascular event^d^* | 370 | **1.95** | **0.02** | 1.14 | 3.32 | 1.70 | 0.06 | 0.99 | 2.91 | 1.68 | 0.06 | 0.98 | 2.88 |
| *Hozpitalization for heart failure^e^* | 99 | *n<5* | | | | *n<5* | | | | *n<5* | | | |
| *Fatal or nonfatal myocardial infarction^f^* | 207 | 1.22 | 0.7 | 0.50 | 2.97 | 1.05 | 0.9 | 0.43 | 2.56 | 1.05 | 0.9 | 0.43 | 2.57 |
| *Fatal or nonfatal ischemic stroke^g^* | 198 | **3.19** | **<0.001** | 1.78 | 5.72 | **2.95** | **<0.001** | 1.63 | 5.34 | **2.87** | **0.001** | 1.58 | 5.20 |
| **Loneliness** |  | **Model 1: Age** | | | | **Model 2** | | | | **Model 3: 2 + Depressive Symptoms** | | | |
|  | *events* | *HR* | *p-value* | *95% CI* | | *HR^h^* | *p-value* | *95% CI* | | *Hri* | *p-value* | *95% CI* | |
| Cardiovascular disease^b^ | 487 | 1.29 | 0.2 | 0.90 | 1.83 | 1.33 | 0.1 | 0.93 | 1.90 | 1.30 | 0.2 | 0.90 | 1.87 |
| Fatal cardiovascular disease^c^ | 83 | **2.52** | **0.004** | 1.34 | 4.77 | **2.43** | **0.008** | 1.27 | 4.68 | **2.33** | **0.02** | 1.18 | 4.60 |
| *Major adverse cardiovascular event^d^* | 370 | 1.42 | 0.08 | 0.96 | 2.10 | 1.45 | 0.07 | 0.98 | 2.15 | 1.42 | 0.09 | 0.95 | 2.13 |
| *Hozpitalization for heart failure^e^* | 99 | 0.87 | 0.8 | 0.35 | 2.14 | 0.89 | 0.8 | 0.36 | 2.20 | 0.86 | 0.7 | 0.34 | 2.14 |
| *Fatal or nonfatal myocardial infarction^f^* | 207 | 1.24 | 0.5 | 0.71 | 2.17 | 1.28 | 0.4 | 0.73 | 2.26 | 1.31 | 0.4 | 0.74 | 2.34 |
| *Fatal or nonfatal ischemic stroke^g^* | 198 | **1.68** | **0.04** | 1.02 | 2.76 | **1.73** | **0.03** | 1.05 | 2.87 | 1.60 | 0.07 | 0.96 | 2.69 |
| **Social Health composite^j^** |  | **Model 1: Age** | | | | **Model 2^h^** | | | | **Model 3^i^: 2 + Depressive Symptoms** | | | |
|  | *events* | *HR* | *p-value* | *95% CI* | | *HR^h^* | *p-value* | *95% CI* | | *HR^i^* | *p-value* | *95% CI* | |
| Cardiovascular disease^b^ | 487 | **1.42** | **0.01** | 1.08 | 1.88 | 1.31 | 0.06 | 0.99 | 1.75 | 1.29 | 0.09 | 0.96 | 1.73 |
| Fatal cardiovascular disease^c^ | 83 | **2.10** | **0.01** | 1.18 | 3.74 | 1.82 | 0.05 | 0.99 | 3.35 | 1.75 | 0.08 | 0.93 | 3.28 |
| *Major adverse cardiovascular event^d^* | 370 | 1.37 | 0.06 | 0.99 | 1.90 | 1.29 | 0.1 | 0.93 | 1.80 | 1.27 | 0.2 | 0.91 | 1.78 |
| *Hozpitalization for heart failure^e^* | 99 | 1.44 | 0.2 | 0.79 | 2.64 | 1.15 | 0.7 | 0.59 | 2.23 | 1.12 | 0.7 | 0.57 | 2.20 |
| *Fatal or nonfatal myocardial infarction^f^* | 207 | 1.26 | 0.3 | 0.80 | 1.99 | 1.18 | 0.5 | 0.75 | 1.86 | 1.19 | 0.5 | 0.75 | 1.90 |
| *Fatal or nonfatal ischemic stroke^g^* | 198 | **1.74** | **0.008** | 1.16 | 2.61 | **1.71** | **0.01** | 1.13 | 2.59 | **1.62** | **0.03** | 1.06 | 2.47 |

^a^ As some end points were composites, a participant who had events for more than one component of the composite (e.g., stroke and then acute myocardial infarction) would contribute only the first event that occurred to the composite end point but would contribute an event to the separate analyses of each component. Hence, summation of the number of events for separate components of a composite end point does not equate to the number of events for the composite end point. If fewer than five participants in a cell, then statistics are not reported to preserve participant’s privacy and potential unreliable statistical inferences.

^b^ CVD incidence, a prespecified secondary end point, was a composite of fatal CHD (death from myocardial infarction, sudden cardiac death, or any other death in which the underlying cause was considered to be CHD), nonfatal myocardial infarction, fatal or nonfatal stroke (including hemorrhagic stroke), or hospitalization for heart failure. 50,887 person-years of observation (mean 4.43±1.28SD years; median 4.51, IQR 3.48-5.53, range 0–7).

^c^ Fatal CVD was defined as any death from stroke (including hemorrhagic stroke) or CHD. 52,353 person-years of observation (mean 4.55±1.21SD years, median 4.61, IQR 3.58-5.60, range 0–7). Fatal CVD assessed for competing events (cancer death, major hemorrhage death, other death).

^d^ Major adverse cardiovascular events, a nonprespecified end point, was a composite of fatal CHD (excluding death from heart failure), nonfatal myocardial infarction, or fatal or nonfatal ischemic stroke. 51,063 person-years of observation (mean 4.44±1.26SD years, median 4.52, IQR 3.48-5.54, range 0–7).

^e^ 51,497 person-years of observation (mean 4.48±1.24SD years, median 4.54, IQR 3.51-5.56, range 0–7).

^f^ 51,297 person-years of observation (mean 4.47±1.25SD years, median 4.54, IQR 3.50-5.55, range 0–7).

^g^ Data for ischemic stroke included cases that were adjudicated as ischemic stroke, cases for which stroke type was uncertain after adjudication, and cases of ischemic stroke with hemorrhagic transformation. 51,349 person-years of observation (mean 4.47±1.25SD years, median 4.54, IQR 3.50-5.55, range 0–7).

^h^ Model 2 adjusted based on a primary CVD risk factors outlined by the Heart Foundation of Australia: age, gender, ethnicity (Caucasian, other), family history of CVD (parents or siblings had a heart attack or stroke), smoking, alcohol consumption (never, former, low risk, and high risk drinking defined as no more than 4 standard drinks on any one day and no more than 10 standard drinks per week(National Health and Medical Research Council (NHMRC), 2020)), physical activity (rarely/never, light, moderate, vigorous), hypertension (yes, no), HDL-c, low-density lipoprotein (LDL; mmol/L), and diabetes.

^i^ Model 3: Model 2 additionally adjused for depressive symptoms (CESD-10 with the lonely item removed, units).

^j^ The social health composite categories were defined as positive (not isolated, supported, and not lonely), or poor (isolated, not supported and/or lonely).

# Appendix 2: Sensitivity Analyses: Social health as a predictor of incident and fatal cardiovascular disease, n=11,486

| **End point** | **Overall** | | **Social isolation** | | | | **Social support** | | | | **Loneliness** | | | | | **Social Health composite^a^** | | | | |
| --- | --- | --- | --- | --- | --- | --- | --- | --- | --- | --- | --- | --- | --- | --- | --- | --- | --- | --- | --- | --- |
|  |  |  | *Hazard Ratio^c^* | *p-value* | *95% Confidence Interval* | | *Hazard Ratio^c^* | *p-value* | *95% Confidence Interval* | | *Hazard Ratio^c^* | | *p-value* | *95% Confidence Interval* | | | *Hazard Ratio^c^* | *p-value* | *95% Confidence Interval* | |
|  | *events* | *events ignored^b^* |  |  |  |  |  |  |  |  |  |  |  |  |  |  |  |  |  |  |
| **Censored first 6mths** |  |  |  |  |  |  |  |  |  |  | |  |  |  |  | |  |  |  |  |
| Incident CVD | 453 | 37 | **1.79** | **0.02** | 1.10 | 2.92 | **2.20** | **0.001** | 1.41 | 3.45 | | **1.45** | **0.04** | 1.01 | 2.06 | | **1.53** | **0.003** | 1.15 | 2.03 |
| Fatal CVD | 83 | 1 | n<5 | | | | n<5 | | | | **2.55** | | **0.004** | 1.34 | 4.83 | | **2.00** | **0.02** | 1.12 | 3.60 |
| **Censored first 12mths** |  |  |  |  |  |  |  |  |  |  | |  |  |  |  | |  |  |  |  |
| Incident CVD | 431 | 87 | **1.78** | **0.02** | 1.08 | 2.95 | **2.21** | **0.001** | 1.40 | 3.51 | | 1.38 | 0.09 | 0.95 | 2.00 | | **1.49** | **0.007** | 1.11 | 2.00 |
| Fatal CVD | 81 | 4 | n<5 | | | | n<5 | | | | **2.59** | | **0.004** | 1.37 | 4.92 | | **1.86** | **0.04** | 1.02 | 3.40 |

If fewer than five participants in a cell, then statistics are not reported to preserve participant’s privacy and potential unreliable statistical inferences.

^a^ The social health composite categories were defined as positive (not isolated, supported, and not lonely), or poor (isolated, not supported and/or lonely).

^b^ Of the events ignored, the number of CVD incident or fatal events prior to the censored dates were: 34/37, 0/1, 56/87, and 2/4

^c^ Adjusted based on a primary CVD risk assessment tool developed specifically from this cohort(Neumann et al., Under review): age (years), gender (women, men), smoking (never, past, current), systolic blood pressure (mmhg), high-density lipoprotein cholesterol (HDL-c; mmol/L), non-HDL (mmol/L), diabetes (yes, no), creatine (mg/dL), and antihypertensive drug use (yes, no).

Fatal CVD assessed for competing events (cancer death, major hemorrhage death, other death).

# Appendix 3: Sensitivity Analyses: : Continuous assessment of social health measures as predictors of cardiovascular disease^a^, n=11,486

1. **Social health responses sequentially numbered**

| **End point** | **Social isolation** | | | | **Social support** | | | | **Loneliness** | | | |
| --- | --- | --- | --- | --- | --- | --- | --- | --- | --- | --- | --- | --- |
|  | *Hazard Radio^h^* | *p-value* | *95% Confidence Interval* | | *Hazard Radio^h^* | *p-value* | *95% Confidence Interval* | | *Hazard Radio^h^* | *p-value* | *95% Confidence Interval* | |
|  |  |  |  |  |  |  |  |  |  |  |  |  |
| Cardiovascular disease^b^ | 0.99 | 0.5 | 0.96 | 1.02 | 0.98 | 0.2 | 0.96 | 1.01 | 1.11 | 0.1 | 0.96 | 1.29 |
| Fatal cardiovascular disease^c^ | 0.97 | 0.3 | 0.90 | 1.03 | 1.01 | 0.7 | 0.95 | 1.08 | 1.24 | 0.1 | 0.93 | 1.67 |
| *Major adverse cardiovascular event^d^* | 1.00 | 0.9 | 0.97 | 1.03 | 0.99 | 0.4 | 0.96 | 1.02 | 1.16 | 0.08 | 0.98 | 1.36 |
| *Hozpitalization for heart failure^e^* | 0.97 | 0.4 | 0.92 | 1.03 | 0.99 | 0.7 | 0.94 | 1.04 | 0.93 | 0.7 | 0.65 | 1.33 |
| *Fatal or nonfatal myocardial infarction^f^* | 1.01 | 0.7 | 0.97 | 1.05 | 0.98 | 0.3 | 0.95 | 1.01 | 1.18 | 0.1 | 0.95 | 1.47 |
| *Fatal or nonfatal ischemic stroke^g^* | 0.99 | 0.6 | 0.94 | 1.03 | 1.18 | 0.1 | 0.95 | 1.46 | 1.18 | 0.1 | 0.95 | 1.46 |

1. **Social health response categories recoded as values**

| **End point** | **Social isolation** | | | | **Social support** | | | | **Loneliness** | | | |
| --- | --- | --- | --- | --- | --- | --- | --- | --- | --- | --- | --- | --- |
|  | *Hazard Radio^h^* | *p-value* | *95% Confidence Interval* | | *Hazard Radio^h^* | *p-value* | *95% Confidence Interval* | | *Hazard Radio^h^* | *p-value* | *95% Confidence Interval* | |
|  |  |  |  |  |  |  |  |  |  |  |  |  |
| Cardiovascular disease^b^ | 1.00 | 0.7 | 0.99 | 1.01 | 1.00 | 0.5 | 0.98 | 1.01 | 1.06 | 0.1 | 0.98 | 1.15 |
| Fatal cardiovascular disease^c^ | 1.00 | 0.9 | 0.97 | 1.02 | 1.01 | 0.4 | 0.98 | 1.04 | 1.12 | 0.2 | 0.96 | 1.30 |
| *Major adverse cardiovascular event^d^* | 1.00 | 0.5 | 0.99 | 1.01 | 1.00 | 0.7 | 0.98 | 1.01 | 1.08 | 0.08 | 0.99 | 1.18 |
| *Hozpitalization for heart failure^e^* | 1.00 | 0.8 | 0.98 | 1.02 | 1.00 | 0.9 | 0.98 | 1.03 | 0.95 | 0.6 | 0.77 | 1.16 |
| *Fatal or nonfatal myocardial infarction^f^* | 1.01 | 0.4 | 0.99 | 1.02 | 0.99 | 0.4 | 0.97 | 1.01 | 1.09 | 0.2 | 0.97 | 1.23 |
| *Fatal or nonfatal ischemic stroke^g^* | 1.00 | 0.9 | 0.98 | 1.01 | 1.00 | 0.9 | 0.98 | 1.02 | 1.10 | 0.09 | 0.98 | 1.24 |

^a^ Adjusted based on a primary CVD risk assessment tool developed specifically from this cohort ^29^: age (years), gender (women, men), smoking (never, past, current), systolic blood pressure (mmhg), high-density lipoprotein (HDL-c; mmol/L), non-HDL (mmol/L), diabetes (yes, no), serum creatinine (mg/dL), and antihypertensive drug use (yes, no). As some end points were composites, a participant who had events for more than one component of the composite (e.g., stroke and then acute myocardial infarction) would contribute only the first event that occurred to the composite end point but would contribute an event to the separate analyses of each component. Hence, summation of the number of events for separate components of a composite end point does not equate to the number of events for the composite end point.

^b^ CVD incidence, a prespecified secondary end point, was a composite of fatal CHD (death from myocardial infarction, sudden cardiac death, or any other death in which the underlying cause was considered to be CHD), nonfatal myocardial infarction, fatal or nonfatal stroke (including haemorrhagic stroke), or hospitalization for heart failure. 50,887 person-years of observation (mean 4.43±1.28SD years; median 4.51, IQR 3.48-5.53, range 0–7).

^c^ Fatal CVD was defined as any death from stroke (including haemorrhagic stroke) or CHD. 52,353 person-years of observation (mean 4.55±1.21SD years, median 4.61, IQR 3.58-5.60, range 0–7). Fatal CVD assessed for competing events (cancer death, major haemorrhage death, other death).

^d^ Major adverse cardiovascular events, a non-prespecified end point, was a composite of fatal CHD (excluding death from heart failure), nonfatal myocardial infarction, or fatal or nonfatal ischemic stroke. 51,063 person-years of observation (mean 4.44±1.26SD years, median 4.52, IQR 3.48-5.54, range 0–7).

^e^ 51,497 person-years of observation (mean 4.48±1.24SD years, median 4.54, IQR 3.51-5.56, range 0–7).

^f^ 51,297 person-years of observation (mean 4.47±1.25SD years, median 4.54, IQR 3.50-5.55, range 0–7).

^g^ Data for ischemic stroke included cases that were adjudicated as ischemic stroke, cases for which stroke type was uncertain after adjudication, and cases of ischemic stroke with haemorrhagic transformation. 51,349 person-years of observation (mean 4.47±1.25SD years, median 4.54, IQR 3.50-5.55, range 0–7).
